# Patient perspectives and preferences for rehabilitation among people living with frailty and chronic kidney disease: a qualitative evaluation

**DOI:** 10.1186/s12882-024-03740-6

**Published:** 2024-09-13

**Authors:** Alice L Kennard, Suzanne Rainsford, Kelly L Hamilton, Nicholas J Glasgow, Kate L Pumpa, Angela M Douglas, Girish S Talaulikar

**Affiliations:** 1 College of Health and Medicine, Australian National University, Canberra, Australia; 2 Department of Renal Medicine, Canberra Health Services, Building 15, Yamba Drive, Garran, Canberra, ACT 2605 Australia; 3 School of Public Health, Physiotherapy and Sports Science, University College Dublin, Dublin, Ireland; 4 Discipline of Sport and Exercise Science, Faculty of Health, University of Canberra, Canberra, Australia

**Keywords:** Kidney disease, Dialysis, Frailty, Rehabilitation, Qualitative

## Abstract

**Background:**

Understanding the patient perspective of frailty is critical to offering holistic patient-centred care. Rehabilitation strategies for patients with advanced chronic kidney disease (CKD) and frailty are limited in their ability to overcome patient-perceived barriers to participation, resulting in high rates of drop-out and non-adherence. The aim of this study was to explore patient perspectives and preferences regarding experiences with rehabilitation to inform a CKD/Frailty rehabilitation model.

**Methods:**

This qualitative study involved two focus groups, six individual semi-structured interviews and three caregiver semi-structured interviews with lived experience of advanced kidney disease and frailty. Interviews were recorded, transcribed, and coded for meaningful concepts and analysed using inductive thematic analysis using constant comparative method of data analysis employing Social Cognitive Theory.

**Results:**

Six major themes emerged including accommodating frailty is an act of resilience, exercise is endorsed for rehabilitation but existing programs have failed to meet end-users’ needs. Rehabilitation goals were framed around return to normative behaviours and rehabilitation should have a social dimension, offering understanding for “people like us”. Participants reported on barriers and disruptors to frailty rehabilitation in the CKD context. Participants valued peer-to-peer education, the camaraderie of socialisation and the benefit of feedback for maintaining motivation. Patients undertaking dialysis described the commodity of time and the burden of unresolved symptoms as barriers to participation. Participants reported difficulty envisioning strategies for frailty rehabilitation, maintaining a focus on the immediate and avoidance of future uncertainty.

**Conclusions:**

Frailty rehabilitation efforts in CKD should leverage shared experiences, address comorbidity and symptom burden and focus on goals with normative value.

**Supplementary Information:**

The online version contains supplementary material available at 10.1186/s12882-024-03740-6.

## Background

Frailty has been characterised as a state of accelerated aging with increased vulnerability to adverse outcomes and non-routine recovery from relatively minor insults [1, 2]. Our previous work exploring frailty in the context of chronic kidney disease (CKD) has demonstrated that frailty is a psycho-emotional-social experience that interacts with comorbidity and symptoms of uraemia [3].Frailty is highly prevalent among patients with advanced CKD, with rates of frailty ranging from 30 to 80% among patients with late-stage CKD and undergoing dialysis, compared to rates of 12% in study participants with stage 3 CKD [4, 5]. Frailty onset occurs at a young age with up to 63% of patients younger than 40 years manifesting frailty at dialysis initiation [6]. As in the general population, traditional risk factors of sarcopenia, inflammation and oxidative stress promote frailty, while CKD-specific factors of uraemia, anaemia, mineral bone disease, impaired nutrition, polypharmacy and dialysis therapy exacerbate its severity [7, 8]. Frailty in this context is associated with various adverse patient outcomes including accelerated CKD progression, worse cognitive impairment and symptom burden, increased risk of hospitalisation, excess infective and cardiovascular complications, reduced access to the benefits of kidney transplantation and death [9–15].

To date there are limited formal guidelines on interventions to maintain or improve functional status in CKD populations. The Kidney Disease Improving Global Outcomes initiative recommends that patients with kidney disease should be encouraged to increase their level of physical activity through exercise training incorporating self-monitoring, verbal reinforcement and motivational support [16]. In a position statement published by the Japanese Society of Renal Rehabilitation, the authors acknowledge the persistent legacy of reports from the 1990’s endorsing rest for patients with CKD, and especially with nephrotic syndrome, where exercise may exacerbate proteinuria and accelerate renal function decline [17]. Contemporary evidence informing this rehabilitation guideline instead supports the use of exercise therapy in CKD, haemodialysis and transplant populations, but remains limited in its examination of impact on hospitalisation, cardiovascular events and mortality outcomes [17]. Likewise, the International Society for Peritoneal Dialysis in collaboration with the Global Renal Exercise Network have recently published practice recommendations for physical activity and exercise in peritoneal dialysis, acknowledging limitations in the quality of evidence and strength of recommendations in the context of frailty with most recommendations denoted 2D (weakly recommended, very little confidence in the estimate of the effect) [18].

Studies of patients undergoing haemodialysis indicate interest in participation in exercise-based programs that offer improvement in strength and fatigue, recognising the implications for mortality and transplant outcomes [19]. One study of US haemodialysis patients reported 98% were concerned that a sedentary lifestyle was unhealthy and endorsed the benefits of physical activity, but only 8% reported no barriers to exercise, describing prevalent limitations of fatigue and shortness of breath [20]. Comorbidity, poor mood, restricted time and limited motivation also limit uptake of exercise interventions in this patient population [21, 22]. Furthermore, clinicians present iatrogenic barriers to appropriate exercise implementation; surveys indicate doctors and nurses believe patient time and adherence is limited and were subsequently less likely to prescribe or offer counselling about exercise [21, 23, 24]. Studies report that clinicians express uncertainty about whether frailty is remediable, with distinctions needed to clarify the distinction between “irreversible” and “reversible” frailty [25, 26]. Rehabilitation remains underutilised in nephrology populations, particularly among those with frailty, despite evidence that intensive inpatient rehabilitation programs offer equivalent improvement in functional outcomes to patients undergoing haemodialysis when compared with non-CKD controls [27, 28]. Evidence supports the use of supervised and longitudinal exercise interventions but is limited by under-representation of patients with CKD/frailty phenotype, recruitment and retention challenges and ability of participants to sustain the activity after study discontinuation [27, 29–33]. Commentators reflect that exercise protocols developed with research priorities in mind have limited utility in engaging patients with multimorbidity and longitudinal care needs [34]. There is a role for consumer engagement and speciality consultation with exercise physiologists to help navigate these barriers.

Understanding the patient perspective of CKD/frailty is critical to offering holistic patient-centred care. Studies increasingly demonstrate that patient activation improves clinical outcomes, enhances patient and staff satisfaction and may reduce health-care costs [35, 36]. For care models to be truly effective they must demonstrate respect for the lived experience, acknowledging the validity of their stories and authority of legitimate patient feedback [35]. This qualitative enquiry seeks to understand patient values, priorities and preferences for exercise intervention in frailty with the view to designing a fit-for-purpose and feasible rehabilitation strategy appropriate to patients with advanced CKD and frailty.

## Methods

This study is reported according to the Consolidated Criteria for Reporting Qualitative Research (COREQ) checklist [37].

### Participants and setting

Participants from the CKD Frailty study [38] with Fried Frailty Phenotype and their caregivers were invited to participate in a focus group or in-depth interview. Eligible participants were English-speaking people with lived experience of advanced chronic kidney disease (defined as eGFR < 20 ml/min) or undergoing maintenance dialysis. People with cognitive impairment defined by existing diagnosis of dementia or Abbreviated Mental Test score ≤ 5/10 were excluded from participation due to inability to provide informed consent. People with functioning kidney transplant were also excluded due to eGFR > 20 ml/min, and the potential confounding impact of immunosuppression. Potential participants were approached by phone or in-person by study investigators. Purposive sampling of diverse patient demographics was utilised. Sample size was informed by the principles of ethnography indicating smaller data collection facilitates in-depth study of the topic, along with principles of theoretical sufficiency which indicate that a homogenous sample usually allows for meaningful analysis and credible conclusions [39–41]. AK provided study information, obtained consent and arranged focus groups. Ethics approval was provided by The Australian National University and The Canberra Hospital Human Research Ethics and Governance Office 2020.ETH.00038. Patient Information and Consent Form was provided. All participants provided informed consent. Consent was documented by signing consent form upon commencement of the interview or verbal agreement where interviews were conducted over phone. Participants were able to withdraw consent up to 2 weeks after conclusion of the focus group discussion, whereupon their discussion contributions would be redacted from the interview transcript. Participants were instructed to respect the privacy and confidentiality of the group and not discuss issues or personal details of the focus group outside of the focus group.

Participants were offered taxi vouchers for participation.

### Data collection

Participant demographic and kidney disease data were collected as part of the larger CKD Frailty study. Demographic details of caregivers were not collected. AK (female, nephrologist, renal supportive care physician and researcher with experience in qualitative research analysis), SR (female, palliative care physician with PhD based on extensive experience in conducting interviews and analysis for qualitative health research) and KH (female, exercise physiologist) conducted two focus groups set within the group education room within the renal outpatients’ area of The Canberra Hospital. Focus group interviews were facilitated to contain a minimum of two and a maximum of six participants per group. Where interested participants were unable to attend a focus group interview, they were offered a one-on-one interview, either in-person or over the telephone, utilising the same open-ended interview approach with AK. Interviews took place between January and April 2023. Interviews were based on Topic Guide developed from literature review on this topic [4, 42]. A pilot interview was conducted to refine the Topic Guide. A Free Association Narrative Interview Method (FANIM) [43] was adopted, recognising that this participative and conversational-style approach is most appropriate in a patient population that may become cognitively tired and require prompts to recall their thoughts [44]. Participants were made aware that the researchers were seeking to understand patient caregiver perspectives of the lived experiences of frailty, participation in frailty rehabilitation and exercise for health maintenance and expectations of care to produce recommendations for frailty rehabilitation strategies in the CKD/Frailty setting. Participants were informed of the researcher’s occupations and research experience and motivations.

During and after each focus group or interview AK, SR and KH documented field notes of key themes that emerged. Focus groups and interviews were audiotaped and transcribed verbatim. The de-identified transcript was coded for meaningful concepts and analysis grouped similar concepts into emergent themes using a constant comparative method of data analysis [45]. Recruitment ceased at data saturation when no new ideas emerged, or additional themes were identified during transcript coding.

Topic guide is available in Supplemental Materials S1.

### Data analysis

Data responses were de-identified before analysis. NVivo12 Plus software [46] was used for thematic analysis. AK read transcripts using inductive thematic analysis, identifying themes at a semantic level, appropriate to health services research to allow a rich and complex account of the phenomenon studied. Analysis followed the steps instructed by Braun and Clark: familiarisation with data, initial coding, searching for themes, reviewing themes using the constant comparison method, monitoring for data saturation, axial coding and finally defining themes for final analysis and report [47]. Social cognitive theory (SCT) was used as a conceptual framework to organise the data and allow participants to relay their experiences through interpretation of their accounts. SCT provides a framework to understand how personal, behavioural and environmental factors interact to influence behaviour and has been used as the analytical framework within other reports of exercise participation in patients with CKD [48]. Concept mapping was used to reflect relationships and linkage between themes. Reliability and validity were ensured through development of a codebook. SR performed coding comparison query using the codebook for rigorous appraisal of themes and meaning. An audit trail documented iteration of themes and analytic decisions.

Clinical trial number: not applicable.

## Results

Twenty-six participants with frailty were approached for participation in focus group discussion or in-depth interviews; twelve participants declined due to competing health concerns including fatigue or fall and clinic burden. Two focus groups (*N* = 4, *N* = 2) and six interviews with participants who had Fried frailty phenotype were conducted, transcribed verbatim and analysed prior to saturation of themes. Three caregiver semi-structured interviews were conducted. Three participants indicated willingness to participate in a focus group but failed to attend: one withdrew due to concerns of privacy and opted for one-on-one interview instead, the other reported he forgot the appointment and subsequently withdrew from participation, the third participant withdrew without providing a reason. Six participants (50%) were dependent on maintenance haemodialysis and 6 (50%) had advanced CKD, one of whom was pursuing conservatively managed non-dialysis kidney care. One participant and one caregiver were directly under the care of AK in her role as nephrologist. One participant undertaking dialysis was directly under the care of AK in her role as renal supportive care physician. All participants had been assessed for frailty by KH. None of the participants were known to SR in a clinical capacity. Focus groups and interviews were up to one hour duration.

Demographic details of participants are provided in Table 1.

**Table 1:** Demographic details of participants

	Participant number	Age (years)	Sex	Modality	Self-described Ethnicity	Caregiver relationship
Individual interviews						
	Patient 1 + Caregiver 1*	79	F	CKD	Welsh	Husband
	Patient 2	72	M	CKD	Australian	N/A
	Patient 3	73	F	HD	Australian	N/A
	Patient 4	88	M	HD	Polish	N/A
	Patient 5	64	M	HD	Australian	N/A
	Caregiver 2	81	F	CKD	Serbian	Daughter
	Patient 6 + Caregiver 3*	63	M	CKD	Australian	Wife
Group 1						
	Patient 7	50	M	CKD	Māori	N/A
	Patient 8	59	F	HD	Greek	N/A
	Patient 9	61	F	CKD	Lebanese	N/A
	Patient 10	72	F	CKD	Australian	N/A
Group 2						
	Patient 11	72	M	HD	Australian	N/A
	Patient 12	74	M	HD	Aboriginal Australian	N/A

*demographic details of caregiver not collected; all patient participants scored > = 3 on Fried Frailty assessment

CKD: chronic kidney disease

HD: haemodialysis

Six themes and 19 subthemes were identified reflecting patient and caregiver perspectives of frailty, kidney disease and rehabilitation. Table 2 provides illustrative quotes.

**Table 2:** Illustrative quotes

Theme and Subtheme	Quote
1) Accommodating and adapting to frailty are acts of frailty resilience	
Equipment and technology	Like, I use the stick vacuum. I can’t use the pull-along one because…It’s too heavy. Also, it gets muddled up with my oxygen [tubing]. (Patient 1) It’s a good flat area [around the house] … There are no steps or anything. Steps are the thing that worry me, unless they’ve got good rails on them that I can hang on to, I can’t walk down steps. I can walk up them, not real well, but I can get up a step. But not down. The place I bought was perfect. It’s a two-storey place. I’ve had a chair lift put in, and I’ve got a bed – the bed cost $8,000. It’s an adjustable bed, do you know the ones that you have in hospital? I keep the place clean. (Patient 2)
Acceptance of limitations	Patient 1: I mean I know my limitations… when I get really puffy… you know, breathing [difficulties] and… sometimes I get a bit wobbling on my feet… You know, then I think maybe it’s time to have a rest for 10 min before I start again. Caregiver 1: I think that’s the biggest thing that – that [she] has been doing, is – is um – she accepts her limitations, gradually. She sets – sets goals to try and get ahead and tries to stay positive. Because of her multiple problems, she could take to her bed and say that’s it. Patient 1: I think you’ve just got to – you’ve really got to help yourself. And only you can do whatever you want to do… no-one else is going to do it for you. So anyway, I just rested, and my daughter was marvellous, and my son and his family were marvellous, and they just carried me along and I’ve slowly just got better. I didn’t feel stressed about it, I just accepted that, you know, what will be will be, and I’ll just keep doing my best. And, um, so here we are today. (Patient 3)
2) Exercise is endorsed for frailty rehabilitation	
Relationship between frailty and inactivity	You know… we’ve got some friends and they think I should sit and not do anything because you’re not well. But I mean I’m not, not well. I feel I’m okay [laughs]… I think you’ve just got to get on with life. But I think the [chair] yoga and walking particularly is really – is really good for you. (Patient 1) And with a sore back, you know, things like that, she is more comfortable sitting at home inside. But I am forcing her. I am trying to get her to go out and do a bit more. (Caregiver 2)
Risk of progression without intervention	I had the stroke 23 years ago and I’m getting worse now than I - I’ve ever been. (Patient 2)
Motivation and internal resources	I need to put myself out and do what I’m asked. Whether it’s diet, exercise, or whatever. (Patient 3) And I really got to get back into it again [laughs]. I think it – I will, yes… you know [the walking] will improve, I’m sure. (Patient 1)
3) Experiences of exercise for rehabilitation: identifying unmet needs	
Non-professional recommendations and self-prescribed programs	I do some exercises. I – I was doing chair yoga but I know how to do that now so I don’t need to put the television on to do it. And I – I do that, you know. It’s… mainly my feet and legs and arms and upper body. It’s not lying down on the floor doing any of [that] [laughs]. If I got down there, I’d never get back up again. A friend of mine that I was having a cup of coffee with, said about it. And she said, have you ever thought about this chair yoga? And I didn’t know anything about it, so I looked on YouTube and there’s about 30 of them on there. So, I picked one that I thought I could do…and then I done it like looking at the television for about two weeks and now I know what to do. I don’t need to sit in front of the television. You know, they have different levels of it there. (Patient 1) Oh, I got one of them cubii things, you know, you pedal. (Patient 12) Well, I got the little pedalling machine here that I do exercise [with]. I’ve got that electric, what do you call it, circulation [machine]. No pain then because I sit down. (Patient 4)
Formal rehabilitation programs are acceptable but have barriers to participation	They were things like, um, walking backwards and forwards. Like the things you asked me to do that day…sitting up, standing and sitting and, you know, and all of that, and weightlifting, and doing - there was a range of things to do that help your muscles. (Patient 3) You only do what you can manage and the nurse or the physiotherapists are monitoring. You learn to take your pulse….the walking might be as far as the person can go. And then it’s measured each time to see if they can go a bit further. So there’s feedback there. (Patient 3) [I went to] the cardiac rehabilitation type thing… with graded exercises to help improve your fitness…. The exercise program itself gets a bit boring after a while…. But the physiotherapists are there to encourage you. They keep making sure that you’re, um… what’s the word? Motivated. You get that positive reinforcement that, um, you’re achieving something. It’s not just sweating for sweating’s sake. (Patient 5) I did have the lady from XX Hospital – must have been last year sometime – ring up. She was the physio from up there – you know, the exercise lady from up there. It was after I went in [to hospital] – after I had all the fluid. But every time she sort of rang up, I was either in hospital or we were going out or something so we never got round to going. (Patient 1) I’ve been to a couple of, uh, exercise places… But it – it – then – it really hurts, like, not hurts me physically, but – but it sort of hurts me, like, doing the exercise, it - it’s so strenuous, you know what I mean? It knocks me up and it’s so uncomfortable, and I hate doing it. (Patient 2) I did go and get a consult…with a, with a physical therapist. And she gave me a list of exercise to do, but I’ve lost it. (Patient 3) I’m not good with being…target’s the wrong word… with being one-on-one. (Patient 5)
4) Rehabilitation goals in frailty are couched in normative behaviours	
Activities of daily living	I look after the pots that I have in the backyard. And I water those normally once a day, but sometimes, if it’s hot, it’s twice a day. I haven’t up to this stage done a lot of extra walking than what I do if I have to go shopping. (Patient 3)
Participation in social spaces	Patient 6: Just like to get fitter. I’d like to get rid of the shakes. I’d like to get stronger again. I’d like to be able to dress myself without having to stop and take a break. Caregiver 3: Take a walk. Patient 6: Visit the park…Socialise with people. Caregiver 3: To be able to be out, living his life again, I think…dealing with everyday life again. We go out for lunch and we go out for dinner sometimes and a bit of shopping – I went and got my hair cut this morning… I just use the wheelie walker for that. I haven’t had to use the motorised scooter. I was walking a lot better than I am now, but I think that will improve as well if I keep doing these things. (Patient 1)
Getting or staying at home	I didn’t want to push myself until I felt confident [that] I could get back home… [Once there] I would get up and do small tasks. I would sit, I would walk outside in the sun. And also, my daughter got a cat, she got a kitten, which was great company. He was a real character. So, I just guess it’s lots of little things that you’re surrounded by that you take joy in… (Patient 3)
5) Frailty rehabilitation and the need to understand “people like us”	
Camaraderie of shared experience	[We need] kind of weekly, um, like physical activity or you know, like group activity. You know, where people like us come together and, you know, help each other and… Look, I went back - I tried to go back to, um, water aerobics, right? But it was just too hard because I think… people don’t realise that you’ve got a medical problem, right, and they just push you - and then I just said to the swimming instructor, I said to her, please don’t push, you know. And she’s like, oh. And after she realised that I had a problem, she’s like, oh, okay, because I had a chat with her after the class. If it was a group of participants of everyone who had kidney disease… See that would be better…we all understand. And I think because everyone’s - we are on dialysis… And people, you know, know each other’s limitations, we all have the same symptoms. (Patient 8) Patient 8: I think this is what we need. I think we need a group that you can chat, you know? And it doesn’t matter whether I’m on dialysis and you are not on dialysis, but you can tell us your experiences. Patient 10: I would really like to, to find out from someone who’s on the dialysis that I’m considering. Patient 7: That would, that would be probably more beneficial than exercise. It’s one thing to know about something, it’s another thing to live it. I’d rather have the group session myself. They [Aboriginal men’s health group] used to take us swimming once a week, you know. And just walking up and down the pool, that sort of stuff like that. But there was always a heap of us. But then they take you out and do these exercises and then give you a big feed after that. (Patient 12) It’s no use doing [rehabilitation] with people that don’t get it. (Patient 9)
Addressing psycho-emotional-social needs	That was what I found was the most positive aspect, apart from the developing your skills and becoming stronger…and you stopped being so fragile-minded, you know. And… really it was the camaraderie of the situation. Some were really scared, and you know. (Patient 3) Patient 8: It needs a social thing as well. Because the physical becomes a social thing. It’s really good because you can connect with each other. Patient 10: And you being on dialysis could give information to others. *Researcher: If you were to design a program for someone with frailty how would you do that?* Patient 3: I think I would first find out how that person is and what they know… and be like a buddy in some ways for them.
Multi-modal program design	Play chess. Exercise your brain… I do a lot of games on my laptop. Yeah, everyday I do my brain exercises. Exercising the brain…. State of mind…I think it’s a lot to do with the state of health. (Patient 4) I would find out what they wanted to know about their condition. I couldn’t get anyone to talk to me. Because we never had kidney disease in my family… we knew nothing about it. I found reading books and things didn’t help me at all because it didn’t mention all the things that I was having trouble with. I like to know and then I feel I’ve got choices about what I can do for myself. Because I believe that we’re as responsible for our health as our doctors are. (Patient 3) A set of exercises which are designed to help, designed for the individual so that they’re not all the same (Patient 5)
Value of consumer design	The person providing [the program] would understand the limitations that you have, especially the um, limitations around your kidney commitment to the dialysis and – and – and the other medical processes. Sometimes I feel that some people, they put together some physical therapy type thing, um, and they think you’re in the army or something. So it needs to come from the viewpoint of the person or persons who are participating in it and an understanding of what their limitations are… designed for the individual so that they – they’re not all the same. (Patient 5) It’s one thing to know about something, it’s another thing to live it. (Patient 10)
6) Barriers and disruptors to frailty rehabilitation in the CKD context	
Unaddressed symptom clusters of fatigue, dyspnoea and pain	I just like to sit here in the afternoon and fall asleep [but when] I go and lie on the bed, I don’t – can’t go to sleep! I think I’m getting to that age now where I need the nana-nap in the afternoons [laughs]. (Patient 1) You know I think we might be twins. [Laughter] I tend to have, uh, a lot of similar issues… I’ve got very poor sleep habit, uh, habits. I can be lethargic, don’t quite have the same amount of energy as I used to… When I was quite young, I used to be quite athletic and quite fit, and used to live quite a [sic] active lifestyle, and I guess as I’ve gotten older, that’s, uh, started to diminish. (Patient 7) The pain that comes in et cetera, and the breathlessness that sort of like discourages you…from doing it [exercise]. (Patient 5) Your body doesn’t have energy. I don’t have energy like before. (Patient 8)
Successful program design should incorporate symptom management	The other thing I guess would be some sort of program to help manage the pain and make joints become more mobile…so, yeah, so that I actually get out and do more walking without actually being in pain all the time. (Patient 5)
Time and energy as a commodities	After dialysis, I come home, and I’m just wrecked… Because your body doesn’t have energy. I don’t have energy like before. (Patient 8) I used to have a bloke come around home and do exercises twice a week, because I only get two days. And you know, you haven’t got any time and that, you got to go to appointments and stuff. Oh, I was just, just having too many doctors’ appointments, so [shrugs shoulders]… Well, the time, you haven’t got that much time you know. (Patient 12) I’d probably do [rehabilitation] on the days that I’m not doing dialysis, right?… Or you know what? It wouldn’t even bother me if I did it in the mornings that, that I don’t do dialysis. I’ll tell you what, when you sit in that chair for four or five hours, you feel like, oh my god, I’ve wasted a whole day doing nothing. (Patient 8) You don’t feel like doing anything…[when] you’re tethered to a machine. (Patient 5) They gave me one of them things, those, you pedal at dialysis… And that was hopeless. I didn’t like that one bit…. Too hard… I said “Nah, I’ll do it at home”. (Patient 12) Because at the moment I’m stuck in the chair for four and half hours. (Patient 5)
Difficulty imagining recovery from frailty	*Interviewer: And what sort of things could you do to try and fix frailty?* Patient 2: Oh, that’s something that I wouldn’t have a clue… If I could think of something, I - I’d try it. Yeah, of course I would. If anything goes wrong at any time… a car, a motorbike, a lawnmower, or anything. I could fix anything during my life, but not now. (Patient 6) But there’s you know, there’s nothing I can do about it at the moment. I know that… Honestly, I don’t know what I could do to improve it (Patient 11)
Desire to maintain the status quo	*Interviewer: if you were to design an exercise program for people like yourself*,* what would it look like?* Patient 1: I think walking. Um. Maybe yoga? What I – what I’ve been doing. That’s been one of my hardest, um, challenges to overcome. The - the future, what does it look like? (Patient 11)

### Accommodating and adapting to frailty are acts of frailty resilience

Participants demonstrated systems for accommodating reduced strength and endurance, along with the requirement for medical equipment. Mastery of the home environment through appropriate use of equipment and technology was linked to self-realisation and an act of resilience in the face of frailty. Individualised and self-sufficient strategies allowed participants to navigate common barriers to exercise participation, suggesting a deeper understanding of frailty and its management. In the absence of evidence-based strategies for frailty intervention, participants with frailty employed internal resources for self-management. Acceptance of one’s limitations emerged as an act of resilience rather than resignation, aligned with perseverance while attending to symptoms and discomfort. Caregivers validated patient’s self-directed efforts for rehabilitation in the face of frailty. Several patients endorsed the role of rest in frailty recovery, suggesting an attitude of passivity, but also great endurance.

### Exercise is endorsed as a means of frailty rehabilitation

Exercise training and physical activity were endorsed by participants as important components of wellbeing and routes to rehabilitation. Participants described social pressures and community expectations of sedentary behaviours but sought to participate in physical activity, demonstrating a deeper knowledge of the relationship of frailty and inactivity. Participants reflected that discussion about the benefits of physical activity prompted uptake of greater physical activity. Participants reported on a legacy of comorbidity and inactivity and identified a growing need for frailty intervention and fear of future debility. In general, participants expressed engagement with rehabilitation strategies and strong internal motivation. Participants demonstrated sustained intention to be more physically active, as well as optimism that states of reduced physical function and frailty could be overcome.

### Experiences of exercise for rehabilitation: identifying unmet needs

Participants were experienced in both formal and informal rehabilitation strategies, with several reporting on non-professional recommendations for physical exercise and demonstrating familiarity with graduated intensity programs as well as progressive confidence and competence. There were disclosed limitations to self-prescribed programs, with the risk of falls and ability to get up off the ground forming a key objective in many formal programs, but dismissed as a goal by participants. Nevertheless, participants exhibited agency, disclosing a variety of self-sourced strategies for maintenance of activity.

Most participants also had experiences with formal rehabilitation and exercise programs, as well as an acceptability of fitness or frailty assessment. Frailty assessment and fitness testing was felt to be acceptable where it facilitated entry to additional care. Programs that incorporated supervision and feedback through a graduated course were felt to be valuable, along with learning strategies for self-monitoring which supported feelings of self-efficacy. Reports of disrupted participation by unpredictable health events were frequent. Experiences of over-vigorous and therefore unsuccessful programs were also common. Programs that lacked social interaction and appropriate support were consistently reported to be ineffective and quickly abandoned.

### Frailty rehabilitation goals are couched in normative behaviours

Activities of daily living emerged as key strategies for maintaining and motivating activity.

Rehabilitation goals were frequently framed around activities of daily living and life participation. Both caregiver and patient participants described a progressive desire for greater independence and social reintegration. Hospitalisation was framed as an event that accelerated frailty or disrupted frailty rehabilitation. Following on from this, return to the home environment after hospitalisation was conceptualised as a key goal wherein activities of daily living could promote recovery and where social and psycho-emotional needs could be met.

### Frailty rehabilitation and the need to understand “people like us”

There was a strong appeal for appropriate understanding from program instructors and the social support and camaraderie made possible by a shared experience of the burden of kidney disease. The “people like us” theme emerged strongly within the focus group setting where participants reflected on the opportunity for social interaction and peer-to-peer education. In the interaction from Focus Group 1 we see participants validate each other’s informational and social needs. Participants articulated a clear preference for formal exercise programs that incorporated social interaction, peer navigation and multi-modality support. Formal group exercise programs were reported to address additional psycho-emotional-social needs and validated the need for interventions for the non-physical aspects of frailty. Critically, participants reported a desire for greater knowledge about their health and disease as a source of agency. Multimodal interventions were envisaged that addressed symptom complaints and were individualised for different needs.

In this way, frailty was conceptualised as a psycho-emotional-cognitive experience as well as a physical state. Participants reported a need for cognitive support and intellectual challenge as part of an effective frailty intervention. Participants strongly endorsed the need for consumer design of rehabilitation programs so that providers were better informed of participants’ individual needs.

### Barriers and disruptors to frailty rehabilitation in the CKD context

Unaddressed symptom burden emerged as a crucial barrier to participation in physical activity, with common experiences of pain, dyspnoea and fatigue/insomnia identified as a key symptom cluster. Participants however, frequently attributed their symptoms to age rather than frailty or kidney disease. Nevertheless, participants identified similarities in symptoms and their disruptive impact on physical activity. Shared experiences of symptom burden emerged as an opportunity to build camaraderie. Pain experiences were very common. Appropriate and adequate pain management strategies were identified as key attributes of an effective rehabilitation program.

The commodities of time and energy were highly valued, particularly by those participants undergoing dialysis. Lack of time was a commonly reported barrier to participation in physical activity, with dialysis and medical appointments dominating. Sometimes this led to activities being ceased. Most participants indicated a preference for physical activity either before dialysis or on a non-dialysis day. The theme of a “wasted day” spent “tethered to a machine” subjugated these preferences. Overall, there was little support for intra-dialytic exercise.

A number of participants reported difficulties envisioning strategies for frailty remediation, nevertheless affirming a commitment to trying. Other participants indicated a sense of nihilism and hopelessness about frailty rehabilitation, revealing a greater burden of cognitive and psycho-emotional frailty. Despite engaging in discussions about rehabilitation, several participants indicated a strong desire for maintaining the status quo. Future planning and goal-setting were described as particularly challenging.

## Discussion

This paper reports a novel and innovative qualitative enquiry into the perceptions, expectations and rehabilitation preferences of patients who are living with advanced CKD and frailty. It also provides unique insights into the roles of caregivers in supporting people with CKD/frailty. Crucially, frailty assessment was found to be acceptable where it offered opportunities to engage in frailty rehabilitation, and a sense of optimism that states of reduced physical function and frailty could be overcome by resilience and recuperation.

### Engagement in frailty rehabilitation

Despite lack of attention by medical systems, patients with frailty and CKD demonstrated interest in frailty rehabilitation through a range of professional and self-sourced strategies. This work dispels the myth that people with frailty are disinterested or poorly engaged in frailty rehabilitation and identifies deeper knowledge of the resilience and recovery strategies employed in the context of frailty. Participants demonstrated insight into the vulnerability posed by frailty, the threat to autonomy and agency, as well as strong desire and demonstrated ability to overcome frailty manifestations. Participants expressed a strong sense of optimism for the possibility of frailty intervention and a commitment to improve physical activity and life participation. Importantly, frailty assessment was found to be beneficial and acceptable when it was felt to offer equitable access to rehabilitation opportunities.

### Key attributes for successful frailty rehabilitation

Within this cohort of patients with CKD and frailty, experiences of formal exercise programs such as cardiac rehabilitation were frequent and informed many of the experiences of recovery. Aligning with the recommendations of many professional bodies, participants endorsed the use of individualised, graduated and supervised programs that offered feedback and motivation [16]. Participants also offered novel insights into key attributes for successful rehabilitation interventions, including a need for social interaction and peer-to-peer support, informational and educational needs, directed by informed and multidisciplinary professionals who were familiar with the CKD/frailty experience. Furthermore, participants endorsed program design that offered self-monitoring and self-sufficiency, reflecting on the safety and confidence that followed observed progress. There was an identified need for longitudinal programs that could accommodate interruptions by hospitalisation and fluctuations in health status characteristic of the CKD/frailty experience. Participants strongly endorsed the use of strategies to improve or maintain cognitive performance. The Japanese Society of Renal Rehabilitation defines renal rehabilitation as “a long-term comprehensive program consisting of exercise therapy, diet therapy and water management, drug therapy, education, psychological/mental support etc. to alleviate physical/mental effects based on kidney disease and dialysis therapy, prolong life expectancy and improve psychosocial and occupational circumstances [17]. Its published guidelines emphasise the importance of addressing the psycho-emotional-social needs of this patient population, recommending a comprehensive, multidisciplinary model that “exhausts all support options to help kidney disease patients smoothly achieve social rehabilitation instead of simply implementing exercise therapy”. Despite this commitment to holistic care, the Guideline remains primarily focussed on exercise therapies. To date few – if any – frailty interventions have offered this holistic and patient-centred model of care, perhaps accounting for the lack of efficacy and limited durability reported to date. Notable for its effect on self-efficacy as well as durable impact 6 months beyond the end of the intervention, Yamaguchi and colleagues’ haemodialysis exercise trial demonstrated considerable flexibility in its program and offers a pilot study for future work in this field [49]. A small pilot study of concurrent exercise training or cognitive challenge utilising tablet-based “brain games” demonstrated that either exercise or cognitive training preserved psychomotor speed and executive function compared to standard care [50]. A further larger randomised controlled trial evaluating the impact of concurrent intradialytic physical and cognitive training has been proposed [51]. We note, however, the focus on intradialytic exercise in these studies, and the strong preferences expressed by our study cohort for inter-dialytic activity. Within the geriatric literature, randomised controlled trial data supports the use of multi-component exercise interventions that incorporate social, nutritional and cognitive elements for effective improvements not only in frailty, but also cognitive performance, emotional support and social networking among frail and pre-frail community-dwelling elderly [52–54]. Crucially, these studies are notable not only for their impressive outcomes, but also their high degree of patient adherence, suggesting that interventions that are fit-for-purpose have high patient acceptability and enhance prolonged participation.

### Symptom burden and impact on participation

Our results have important implications unique to the care of patients with advanced kidney disease and frailty. In our study, we describe high rates of CKD/frailty symptomatology which act as a chief barrier to physical activity. Participants frequently reported pain, dyspnoea, fatigue, exhaustion and disturbed circadian rhythm. This report of patient experiences aligns closely with patient perspectives revealed by the SONG initiative, including the debilitating and exhausting burden of dialysis, the cycle of post-dialysis exhaustion, inhibited rest, lack of remedy or relief from symptom burden, restricted life participation, diminished relationship roles and dependence on others [55]. While many participants demonstrated resilient strategies to overcome these impediments, successful rehabilitation strategies must incorporate symptom assessment and management into their care models. This calls for a range of multidisciplinary and experienced clinicians who are willing to engage not only in frailty and rehabilitation, but also pain and symptom management as well as addressing psycho-emotional-social needs. A collaborative healthcare model should include nephrologists, renal supportive care physicians and pain specialists, rehabilitation therapists and exercise physiologists, dietitians, nursing specialists, social workers, psychologists, occupational therapists and pharmacists to truly meet the needs of this patient population.

### Consumer design and peer support needs

Just as the SONG initiative prioritises patient perspectives, our study utilises consumer engagement to inform the design of an innovative frailty intervention. Our qualitative methodology recognises consumers as experts in their own care and active participants in the delivery of successful frailty rehabilitation. Participants recommended the use of peer-to-peer support and education, deriving benefit from lived experienced and social engagement. Recent systematic review suggests that exercise programs involving peers can promote and maintain adherence to exercise programs [56]. Participants reflected that discussion about physical activity promoted uptake of physical activity, identifying a key role for clinicians as well as peer navigators. Future work should also explore how clinicians might seek to reinforce intrinsic motivation, which has been shown to predict long-term engagement with physical activity, including in chronic pain settings [57, 58].

### The role of caregivers in frailty rehabilitation

This study also offers novel and valuable insights into the role of caregivers as key support people in promoting frailty recovery. Caregivers were seen to validate the lived experience of frailty and advocate for the need for frailty interventions. Caregivers also emerged as strategic goal-setters, endorsing life participation and social re-integration. Future work should explore how frailty intervention impacts caregiver burden and wellbeing.

### Limitations and reflexivity

The findings of this study should be interpreted with some caution. The views of participants reported here reflect the perspectives and preferences of a cohort of patients attending a single Metropolitan centre. Participants with functioning kidney transplant and those undertaking peritoneal dialysis were not represented in our study cohort and thus findings may not be generalised to these patient groups. While methodological approaches allowed purposive sampling of diverse patient demographics, cultural understandings of frailty and priorities for care remain to be fully understood. Primary investigator AK is a member of staff at the research setting and thus shared a clinical relationship with some of the participants through either nephrology clinics or renal supportive care encounters. It is possible that participants, either consciously or subconsciously, felt an expectation that by participating in discussion about rehabilitation options, that they would be able to access rehabilitation and additional care. This speaks to the moral imperative of researchers in this field to engage in and commit to interventional, not just observational, work. The key attributes of a frailty intervention offered by this work should allow development and inform the design of future interventional trials for frailty incorporating flexible group exercise training not limited to intradialytic exercise.

## Conclusions

This study offers key attributes for successful implementation of frailty rehabilitation in the CKD context. To our knowledge, this is the only existing study to explore patient and caregiver perspectives and priorities for frailty remediation. Our example of patient activation promises opportunity to codesign a durable fit-for-purpose intervention for frailty that addresses psycho-emotional-social needs alongside symptom burden and physical frailty. Frailty assessment was found to be acceptable where it afforded opportunity to engage in self-directed care. We reveal a range of resilience strategies alongside a strong sense of optimism for frailty recovery shared by participants. Healthcare providers and policy writers must embrace the possibility of frailty recovery. Until there is evidence to the contrary, we owe it to our resourceful patients to thoroughly investigate frailty interventions.

## Electronic supplementary material

Below is the link to the electronic supplementary material.

Supplementary Material S1: Topic Guide for focus groups and semi-structured interviews

## Data Availability

Data is provided within the manuscript and supplementary information. Full data set can be made available upon reasonable written request to the corresponding author.

## Abbreviations

CKD: Chronic kidney disease
HD: Haemodialysis
COREQ: Consolidated Criteria for Reporting Qualitative Research
FANIM: Free Association Narrative Interview Method
SCT: Social cognitive theory
HD: Haemodialysis
