# Supplementary material for: Patient perspectives and preferences for rehabilitation among people living with frailty and chronic kidney disease: a qualitative evaluation
Source: BMC Nephrol. 2024 Sep 13;25:304. doi: 10.1186/s12882-024-03740-6 (PMC11401252; doi:10.1186/s12882-024-03740-6)
Supplement: Supplementary file 1 — Supplementary Material S1: Topic Guide for focus groups and semi-structured interviews [file 12882_2024_3740_MOESM1_ESM.docx]

Supplemental Materials

S1 Topic Guide for focus groups and semi-structured interview

- Experiences of frailty and reduced physical ability
- “How do you feel about your current state of health or fitness?”
- “What does the word frail mean to you?” “What does a frail person look like to you?”
- “How has frailty influenced your activities?”
- “How has frailty influenced your decisions?”
- “How has frailty influenced your plans for the future?”
- Experiences of frailty screening
- How do you feel about being assessed for frailty?
- Perceived barriers to physical activity
- “What are the barriers to physical activity for you?”
- “What are the problems with doing physical activity?”
- Perceived facilitators to physical activity
- What sorts of things make physical activity easy or better?”
- “How could you be helped to do more physical activity?”
- Preferences for physical activity
- ”What sort of physical activity do you like to do?”
- “What sort of physical activity would you like to do?”
- “If you were to design an exercise program for people like yourself, what would that look like?”
- “If you had the power to change things about your physical activity, what would you change?”
- “When would you like to do physical activity?”
- “Think of someone you know who is frail but wanted to become fitter/stronger. How would you help that person?”
- Expectations following a rehabilitation program
- “What would be your hopes if you were to participate in a physical reconditioning program?”
- “What would you like to see happen if you did more physical activity?”

Prompts and invitations:

- That’s very helpful, thank you.
- You’ve given me a lot of good information there.
- I appreciate your willingness to share your experience. Every experience is unique, and we’ve heard all kinds of things. There’s no wrong or right answer to any of these questions. What matters is that it’s your experience.
- It would be helpful to hear more about that.
- I can see that question evokes strong emotions. Take your time, or if you’d like, we can change topics and come back to this later.

Signalling transitions:

- Thank you for your thoughts about frailty, let’s talk now about how we might eb able to make things better.
- Let me turn now to your personal likes and dislikes about physical activity.
- We’ve been talking about your past experiences of physical activity. The next set of questions is about your preferences for future activity.
